# Phenomenology or constructivism in psychopathology

**DOI:** 10.1192/j.eurpsy.2024.1371

**Published:** 2024-08-27

**Authors:** G. Dzhupanov

**Affiliations:** State psychiatric hospital for treatment of drug addiction and alcoholism, Sofia, Bulgaria

## Abstract

**Introduction:**

Phenomenology is historically fundamental for psychopathology. In recent decades constructivist approaches occur as an alternative. Some consider them quite compatible, others take the reverse stance, arguing for advances of one or the other. This has parallel in discussions and contradictions in philosophy of mind.

**Objectives:**

As Dennett points, there is no science free of philosophy, so it is recommendable to make clear and bear in mind on what kind of philosophy is based contemporary psychopathology.

**Methods:**

Brief review and comparison between phenomenological and constructivist approaches.

**Results:**

There is no doubt, that culture influences self and experience. Culture and social environment shape abnormal experiences as well. In an extreme variant a constructivist statement would sound as “Someone suffers from a disorder because a violation of social norms.” The self is considered as socially constructed entirely, in the spirit of Mead. Psychopathological theories are function of societal development as well. Phenomenological approach pays attention to constitution and structure of subjective experience. The self has a multilayer structure with a pre-reflexive experiential level of self. Elements of subjective reality do exist, that are not result from social influence, these include abnormal experiences. Especially some experiences in severe mental illness originate from profound disturbance of intentionality based on dysfunction of pre-reflexive self-awareness as it shown by T. Fuchs.

**Conclusions:**

Phenomenology offers more broad and satisfying framework for psychopathology and psychiatry. Contribution of constructivism is not to be ignored, but seems to be one-sided. Further research and deeper education in phenomenological psychopathology of trainees would be valuable.

**Disclosure of Interest:**

None Declared

